# Novel and Recurrent Mutations in a Cohort of Chinese Patients With Young-Onset Amyotrophic Lateral Sclerosis

**DOI:** 10.3389/fnins.2019.01289

**Published:** 2019-12-06

**Authors:** Jianwen Deng, Wei Wu, Zhiying Xie, Qiang Gang, Meng Yu, Jing Liu, Qingqing Wang, He Lv, Wei Zhang, Yining Huang, Tao Wang, Yun Yuan, Daojun Hong, Zhaoxia Wang

**Affiliations:** ^1^Department of Neurology, Peking University First Hospital, Beijing, China; ^2^National Institute of Biological Sciences, Beijing, China; ^3^Department of Neurology, The First Affiliated Hospital of Nanchang University, Nanchang, China

**Keywords:** amyotrophic lateral sclerosis, young-onset, fused in sarcoma, novel mutation, c.1528A > C (p. K510Q), Drosophila model

## Abstract

Amyotrophic lateral sclerosis (ALS) is a progressive neurodegenerative disease that affects nerve cells in the brain and spinal cord. More than 25 ALS-related genes have been identified, accounting for approximately 10% of sporadic ALS (SALS) and two-thirds of familial ALS (FALS) cases. Several recent studies showed that genetic factors might have a larger contribution to young-onset ALS than to ALS cases overall. However, the genetic profile of young-onset ALS patients is not yet fully understood. Here, we investigated a cohort of 27 young-onset ALS patients (onset age < 45 years) through whole-exome sequencing (WES). Genetic analysis identified pathogenic variants of *FUS* (25.9%), *SOD1* (22.2%), *TARDBP* (3.7%), and *VCP* (3.7%) in 27 young-onset ALS patients. Of 12 identified types of mutations, c.1528A > C in *FUS* and c.266G > A in *VCP* were novel. All of the cases in this study reflect a monogenic origin with an autosomal dominant mode of inheritance. Notably, a novel *de novo* missense mutation, c.1528A > C (p.K510Q), in *FUS* was identified in a 29-year-old ALS patient. Expression of the K510Q mutant FUS resulted in cytoplasmic mislocalization of FUS in cultured cells and induced neural toxicity in a fly model. This study provides further evidence of the genetic profile of young-onset ALS patients from China and expands the mutational spectrum of the *FUS* gene, with one new K510Q mutation identified.

## Introduction

Amyotrophic lateral sclerosis (ALS) is a fatal neurodegenerative disease affecting the upper and lower motor neurons of the brain and spinal cord, mainly characterized by weakness, muscle atrophy, dysarthria, and breathing difficulties (Rowland and Shneider, 2001). About 10% of ALS patients have a family history, whereas 90% are classified as sporadic (Riva et al., 2016; Chia et al., 2018). Since superoxide dismutase 1 (*SOD1*) was reported as the first ALS-associated gene in 1993 (Rosen et al., 1993), more than 25 genes have been described to be associated with ALS, including TAR DNA-binding protein 43 (*TARDBP*), fused in sarcoma (*FUS*), valosin-containing protein (*VCP*), and *C9ORF72* (Nguyen et al., 2018). Mutations in these genes have been identified in approximately two-thirds of familial ALS (FALS) and 10% of sporadic ALS (SALS) cases (Chia et al., 2018). In a Chinese ALS cohort, less than 40% of FALS and 6.5% of SALS were identified to have validated mutations. While mutations of *SOD1* were the most frequent in FALS (30.6%), mutations were second-most frequent (5.6%) in *FUS*, and this was also the most abundant mutation in SALS (1.7%) (Liu et al., 2016).

Several recent studies have shown that *FUS* gene mutations might contribute more to young-onset ALS than other gene mutations (Zou et al., 2013; Corcia et al., 2017; Gromicho et al., 2017). In a German cohort, mutations in *FUS* were identified in six out of 14 SALS patients (age of onset < 35 years old). Similarly, five out of 12 juvenile Chinese ALS patients carried mutations in the *FUS* gene (Liu et al., 2017). However, there have only been a few reports on the frequency of gene mutations in young-onset ALS patients whose age of onset was less than 45 years old (Turner et al., 2012). Thus, the characterization of the mutational spectrum in young-onset ALS for genetic counseling and diagnosis is highly relevant. Herein, we systematically evaluated the frequency of ALS-associated gene mutations in a cohort of young-onset ALS. We identified mutations in several genes, including a novel *de novo* K510Q FUS mutation. To examine if K510Q FUS mutation was pathogenic, K510Q mutant FUS protein was expressed in cultured cells and a transgenic fly model to study its cellular distribution and neural toxicity.

## Materials and Methods

### Subjects

All patients in this study were diagnosed as having definite or probable ALS according to the EI Escorial criteria (Brooks et al., 2000) and were enrolled in the Department of Neurology of Peking University First Hospital, China, from January 2015 to December 2018. An additional criterion was that the age of onset should be less than 45 years. The definition of ALS was based on the presence of at least one first-degree or second-degree relative with similar symptoms, as it is defined in previous studies (Liu et al., 2016; Muller et al., 2018). All clinical materials used in this study were obtained for diagnostic purposes with written informed consent. The study was approved by the Ethics Committee of the First Hospital of the PKU 2012[542].

### Genetic Testing

After informed consent, patient blood samples were collected. Genomic DNA was extracted from peripheral blood using a MagPure Blood DNA Midi KF Kit according to the manufacturer’s instructions (Magen, China). Whole-exome sequencing (WES) was performed. Multiple neurodegenerative genes including 27 known ALS genes (*SOD1, ALS2, SETX, FUS, VAPB, ANG, TARDBP, FIG 4, OPTN, VCP, UBQLN2, KIF5A, TIA1, ANXA11, SIGMAR1, CHMP2B, PFN1, C9ORF72, ATXN2, AR, DCTN1, NEFH, PRPH, DAO, TFG, TAF15*, and *GRN*) were analyzed. Hexanucleotide repeat expansion in *C9orf72* was monitored by repeat-primed polymerase chain reaction (RP-PCR) according to the literature (Cleary et al., 2016). In brief, RP-PCR reactions in a total volume of 20 μl consisted of 0.8 × Optimized DyNAzyme^TM^ EXT buffer, 0.16 mM dATP, 0.16 mM dTTP, 0.56 mM dCTP, 0.56 mM dGTP, 1.8 M Betaine, and 0.12 U/μl DyNAzyme^TM^ EXT DNA Polymerase (ThermoFisher Scientific). The primer concentrations were 0.5 μM FAM-labeled F, 0.25 μM repeat, and 0.75 μM Tail R, and 200 ng DNA was added. The primer sequences used here have been reported previously (Cleary et al., 2016). After incubation at 94°C for 7 min, the following cycling sequence was performed: 35 cycles of 95°C for 45 s, 98°C for 10 s, 62°C for 30 s, and 78°C for 6 min (slow ramp rate of 0.6°C/s), then a final elongation step of 78°C for 10 min. PCR products were separated by capillary electrophoresis using an ABI 3130×L with a 50 cm array (Life Technologies) with either Genescan^TM^ LIZ600 or LIZ1200 size standard (Life Technologies). Data were analyzed using GeneMarker^®^ software v2.4.0 (Soft Genetics). The Human Gene Mutation Database^1^, the ClinVar database^2^, the Pubvar Database^3^, and the public polymorphism database^4^ were consulted to determine clinical significance.

### Functional Study

#### Plasmids

The enhanced green fluorescent protein (EGFP)-tagged pEGFP-N1-FUS Wt, P525L, and K510Q plasmids were constructed as in a previous study (Deng et al., 2015). Briefly, the Wt or mutant FUS was amplified using PCR and subcloned into the pEGFP-N1 vector.

#### Cell Culture and Transfection

HEK293 or HT22 cells were cultured in DMEM (Gibco) supplemented with 10% fetal bovine serum (FBS) (Gibco), and 100 unit/ml penicillin-streptomycin in a humidified incubator at 37°C under 5% CO_2_/95% air. Transient transfection was performed using Lipofectamine 3000 (Invitrogen) following the manufacturer’s instructions.

#### Fluorescence Microscopy

HEK293 or HT22 cells were rinsed with 1X PBS, fixed with 4% polyformaldehyde in 1X PBS at 48 h post-transfection with empty vector (Ctr) or Wt, P525L, or K510Q FUS-EGFP. The samples were mounted using prolong gold antifade mounting medium with DAPI (Invitrogen). Images were acquired at 60 × magnification by using an ECLIPSE Ti2-E (Nikon) inverted research microscope.

#### Fly Strains and Cultivation

Fly stocks were cultivated on standard food and kept at 25°C. The GMR-Gal4 (Bloomington Drosophila Stock Center, BDSC) strain in this study was used in previous studies (Chen et al., 2011). Generation of UAS-K510Q-FUS transgenic flies was performed as follows. The K510Q mutant FUS cDNA was cloned into the attB-pUAST vector, sequence-verified, and subsequently inserted into attP40 or attP2 sites of phiC31 stocks via standard microinjection. Both insertion lines produced a similar effect in this study.

#### Western Blot Analysis

Fly eye tissues were lysed with RIPA buffer (1% NP-40, 0.5% sodium deoxycholate, 0.1% SDS, pH7.4) containing a cocktail of protease inhibitors (Roche). Cell lysates were analyzed by Western blotting using the corresponding specific antibodies to detect FUS and Elav proteins. The pan-neuronal marker Elav was used as a loading control as previous reported (Wang et al., 2019).

## Results

### Genetic Results in This Cohort of Young-Onset ALS Patients

**FIGURE 1:**
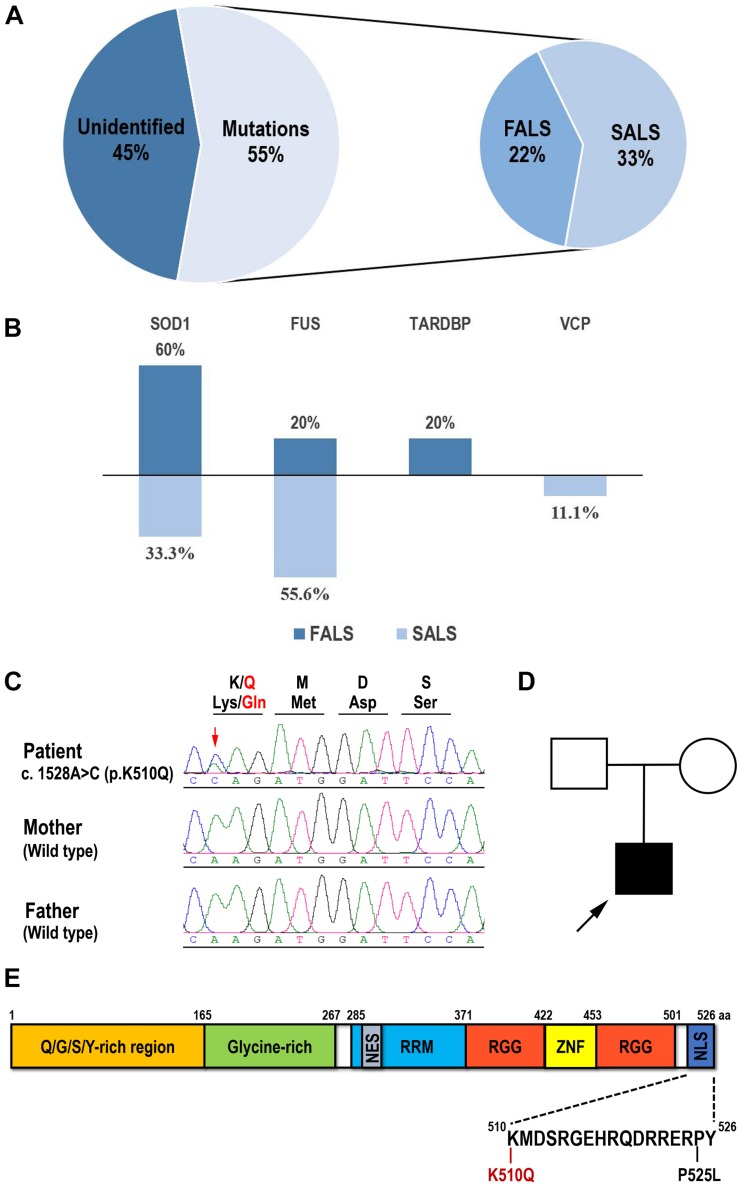
Mutation spectrum of Chinese young-onset ALS and identification of the K510Q FUS mutation. **(A)** Pie chart showing that 55% of young-onset ALS patients had identified validated mutations, consisting of 22% familial ALS (FALS) and 33% sporadic ALS (SALS). **(B)** Percentage of each validated gene in FALS and SALS in this cohort. *SOD1* has the highest mutation frequency in FALS and *FUS* in SALS. **(C)** Sequencing results of amplified genomic DNA from the patient and his parents. The novel FUS mutation c.1528A > C (p. K510Q) was identified in the patient. Mutated nucleotide was indicated by arrowhead. The reading frame depicting the corresponding amino acid substitution is shown on the top of the electropherogram. **(D)** The family pedigree of the patient with ALS. The proband was indicated by arrowhead. **(E)** Schematic diagram of the full-length human FUS protein and the K510Q and P525L mutations located in the nuclear localization signal domain (NLS).

In this study, we recruited a cohort of 27 unrelated young-onset ALS patients (Patient 1 to 27), including seven FALS patients and twenty SALS patients. A total of 12 variants were identified in 15 of the 27 patients, consisting of six FALS (6/7, 85.7%) and nine SALS (9/20, 45%) patients (Figure 1A). Seven patients (7/27, 25.9%) carried *FUS* mutations, six patients (6/27, 22.2%) carried *SOD1* mutations, one patient (1/27, 3.7%) carried a *TARDBP* mutation, and one patient (1/27, 3.7%) carried a *VCP* mutation (Figure 1B). No pathogenic repeat expansion in *C9orf72* was identified. These mutations were regarded as pathogenic or likely pathogenic according to ACMG variant classification criteria except for a novel p.K510Q *FUS* mutation. *SOD1* mutations were the most frequent in FALS (3/7), whereas *FUS* mutations were the most frequent in SALS (5/20) (Figure 1B). Available Sanger sequencing confirmed that the P525L *FUS* variant in Patient 2 and R89Q *VCP* variant in Patient 4 are *de novo* variants. The G142A variant in *SOD1* was segregated in the family of Patient 23. Only one pathogenic variant was identified in genes associated with ALS in each of these patients. In this study, we tested the pathogenicity of the variant K510Q in *FUS*, which had never been reported previously.

### Clinical Features of This Cohort of Young-Onset ALS Patients

An overview of the clinical data of all of the patients is shown in Table 1. Out of seven familial ALS probands (three males and four females), three patients developed limb weakness and another patient developed dysphagia as the first symptom. Out of 20 sporadic ALS cases (eleven males and nine females), 17 experienced weakness of extremities, and three developed dysphagia or dysarthria initially. In our study, the median disease duration could not be estimated, as some patients were still alive at the end of the study and the disease duration of some patients was not available.

**TABLE 1:** Clinical and genetic data on 27 young-onset ALS patients.

**Patient**	**Gender**	**Age of onset (y)**	**Location of onset**	**Family history**	**Genetic variant**	**Clinical significance**	**Disease duration (months)**
1	F	16	Spinal	SALS	*FUS*, c.1574C > T, p.P525L	Pathogenic	NA
2	M	19	Bulbar	SALS	*FUS*, c.1574C > T, p.P525L	Pathogenic	NA
3	M	22	Spinal	FALS	*FUS*, c.1574C > T, p.P525L	Pathogenic	8 (alive)
4	M	24	Spinal	SALS	*VCP*, c.266G > A, p.R89Q^#^	Likely pathogenic	5
5	F	25	Bulbar	SALS	*FUS, c.1483C* > *T*, p.R495^#^	Pathogenic	20
6	M	27	Spinal	FALS	*FUS*, c.1562G > T, p.R521L	Pathogenic	NA
7	F	27	Spinal	SALS	not identified	–	46 (alive)
8	F	28	Spinal	SALS	not identified	–	60 (alive)
9	M	29	Spinal	SALS	*FUS*, c.1528A > C, p.K510Q^#^	Uncertain	15 (alive)
10	M	32	Spinal	SALS	not identified	–	NA
11	F	33	Bulbar	FALS	*TARDBP*, c.1132A > G, p.N378D	Pathogenic	NA
12	F	34	Bulbar	SALS	not identified	–	NA
13	M	36	Spinal	SALS	not identified	–	75 (alive)
14	F	36	Spinal	FALS	not identified	–	10 (alive)
15	F	38	Spinal	SALS	not identified	–	23 (alive)
16	F	38	Spinal	SALS	not identified	–	19 (alive)
17	M	38	Spinal	SALS	*SOD1*, c.125G > A, p.G42D	Pathogenic	8 (alive)
18	M	39	Spinal	SALS	not identified	–	60 (alive)
19	M	39	Spinal	SALS	not identified	–	NA
20	F	41	Spinal	FALS	*SOD1*, c.425G > A, p.G142E	Pathogenic	47 (alive)
21	F	42	Spinal	SALS	*SOD1*, c.124G > A, p.G42S	Pathogenic	38 (alive)
22	M	42	Spinal	SALS	*FUS*, c.1562G > A, p.R521H	Pathogenic	9 (alive)
23	M	42	Spinal	FALS	*SOD1*, c.425G > C, p.G142A	Pathogenic	14 (alive)
24	F	42	Spinal	SALS	not identified	–	12 (alive)
25	F	44	Spinal	FALS	*SOD1*, c.125G > A, p.G42D	Pathogenic	20 (alive)
26	M	44	Spinal	SALS	*SOD1*, c.436G > A, p.A146T	Pathogenic	15
27	M	45	Spinal	SALS	not identified	–	12 (alive)

*Abbreviations: SALS, sporadic amyotrophic lateral sclerosis; FALS, familial amyotrophic lateral sclerosis; *FUS*, fused in sarcoma; *TARDBP*, TAR DNA-binding protein 43; *SOD1*, superoxide dismutase 1; *VCP*, valosin-containing protein; ^#^novel mutation; NA, not available.*

Six patients carrying *SOD1* variants suffered from the disease at the ages of 38, 41, 42, 42, 44, and 44 years, respectively. Half of them were FALS patients. Five out of six patients developed motor symptoms in the lower limbs. Patient 4, carrying a p.R89Q *VCP* variant, died of respiratory failure about 5 months after the initial limb weakness. Patient 11, carrying a p.N378D *TARDBP* variant and whose 24-year-old sister had died from similar symptoms, developed dysphagia at the age of 33 years old. Patient 18 received a tracheostomy in the third year of onset and had no identified pathogenic variant.

Seven patients carrying a *FUS* mutation (five males and two females) developed first symptoms at the ages of 16, 19, 22, 25, 27, 29, and 42 years, respectively. Patients 2 and 6 had a bulbar onset, and Patients 1 and 9 developed predominant bulbar symptoms in the course of the disease.

The patient carrying the novel p.K510Q mutation developed weakness of his right arm at the age of 29 years old, which was followed by whole limb weakness and dysphagia in a month. On examination, he presented tongue atrophy, weakness of neck flexion and extension, and extensive weakness of upper and lower extremities. There was muscle atrophy in the upper extremities, with bilateral Hoffman sighs, and increased tone in the lower extremities, with elevated tendon reflexes and bilateral positive ankle clonus. By 6 months, he had evidence of acute and chronic denervation on EMG testing in the upper extremities, lower extremities, and sternocleidomastoid muscles. He suffered from respiratory difficulties about 15 months after the first spinal symptom and received invasive ventilation therapy. He had no remarkable family history. Sanger sequencing confirmed the presence of the c.1528A > C (p. K510Q) *FUS* heterozygous variant in this patient and its absence in his parents (Figures 1C,D).

### Functional Analysis of p.K510Q *FUS* Mutation

#### Cellular Model: K510Q FUS Mislocalized in Cytoplasmic Inclusions in HEK293 and HT22 Cells

**FIGURE 2:**
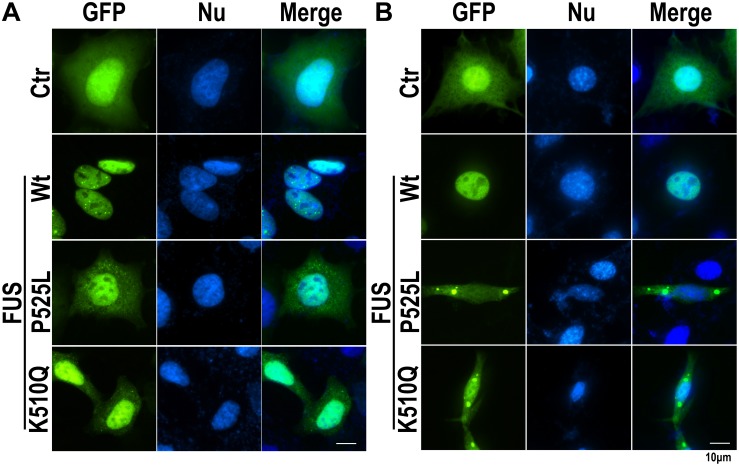
The K510Q mutant FUS is mislocalized in cytoplasm in HEK293 and HT22 cells. **(A)** HEK293 cells were transfected with EGFP-tagged Wt, P525L, or K510Q FUS. P525L or K510Q mutant FUS proteins were mislocalized in the cytoplasm. **(B)** Mouse neuronal cells (HT22) were transfected with EGFP-tagged Wt, P525L, or K510Q FUS. Cytoplasmic inclusions of mutant FUS were found in cells expressing P525L or K510Q mutant FUS. Scale bar: 10 μm.

Since the K510Q mutation was located in the nuclear localization signal (NLS) of the FUS protein (Figure 1E), we first investigated whether this mutation caused FUS mislocalization in cultured mammalian HEK293 cells compared with wild type (Wt) or P525L FUS. Wt FUS localized in the nucleus, whereas the P525L mutant mislocalized from the nucleus to the cytoplasm. The K510Q mutant also caused FUS cytoplasmic localization, although the majority of the mutant protein was still retained in the nucleus (Figure 2A).

We further examined the subcellular localization of K510Q mutant FUS in a mouse neuronal cell line (HT22 cells). While Wt-FUS localized in the nucleus, the K510Q mutant was largely localized in the cytoplasm of neuronal cells and formed inclusions, similar to the P525L mutant (Figure 2B). The results from both HEK293 and HT22 neuronal cells consistently indicated that the novel K510Q mutation could be the pathogenic cause of ALS.

#### Animal Model: K510Q Mutant FUS Caused Retinal Degeneration in Transgenic Fly Eyes

**FIGURE 3:**
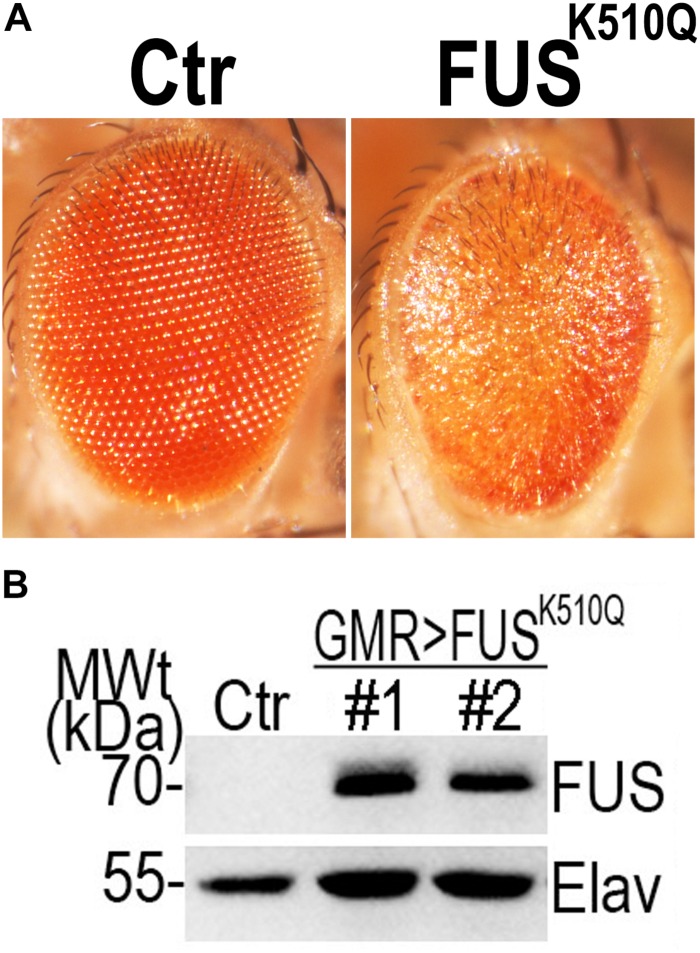
Over-expression of the K510Q mutant FUS in retinal neuron induced neural toxicity in a transgenic fly model. **(A)** Microscopic images of fly eyes expressing Ctr and K510Q mutant FUS. Fly genotypes: Ctr: GMR-Gal4/W1118; FUS^K510Q^: GMR-Gal4/UAS-K510Q-FUS. **(B)** Western blot analysis of FUS protein in transgenic fly eyes. The pan-neuronal marker Elav was used as a loading control.

Since the expression of mutant FUS in transgenic fly eyes can be used to analyze the neural toxicity of mutant FUS protein (Chen et al., 2011; Deng et al., 2015, 2018), we generated transgenic flies expressing Ctr or K510Q mutant FUS under the GMR-Gal4 driver. Flies expressing K510Q mutant FUS in photoreceptors exhibited retinal degeneration, with reduced pigmentation and a loss of ommatidia (Figure 3A). This phenotype was reported in previous studies (Chen et al., 2011, 2016; Deng et al., 2015). Correspondingly, the expression of K510Q mutant FUS was confirmed by Western blot analysis (Figure 3B). These results indicate that K510Q mutant FUS induced neural toxicity in ALS.

## Discussion

Genetic studies have enabled great progress and provided insights into the complex pathogenic mechanisms leading to motor neuron degeneration. So far, at least 25 ALS-associated genes have been reported (Nguyen et al., 2018). Here, we evaluated the frequency of pathogenic variants in ALS-associated genes in young-onset Chinese patients. In our cohort, *SOD1* was the most frequently mutated gene in familial young-onset ALS patients, while the *FUS* gene was the most frequently mutated in sporadic young-onset ALS patients. These data are consistent with another study on the mutation spectrum in Chinese ALS patients reporting more than 200 cases (Liu et al., 2016). In patients with *SOD1* variants, the median age of onset (42, 38–44) is higher than for other genes, and the motor symptoms begin in the lower limbs. These characteristics are consistent with previous reports (Sabatelli et al., 2013; Mathis et al., 2019). Patient 4 in this study, carrying the p.R89Q *VCP* variant, showed earlier onset and more aggressive progress in comparison with patients carrying other *VCP* variants in the previous study (Koppers et al., 2012). The p.N378D TARDBP variant had a familial inheritance pattern, which is similar to the previous study, but Patient 11 in this study, carrying the p.N378D TARDBP variant, showed early onset and an initial bulbar symptom at different locations of onset (Tsai et al., 2011). *De novo* mutations in the *FUS* gene are the most frequent genetic cause in early onset ALS patients (Zou et al., 2013; Hubers et al., 2015; Kim et al., 2015), indicating that *FUS* mutations are associated with early onset and with a reduced life expectancy and reduced reproductive fitness.

FUS proteinopathy is present in a group of heterogeneous disorders, including amyotrophic lateral sclerosis (ALS-FUS) and frontotemporal lobar degeneration (FTLD-FUS), which are both characterized by the formation of inclusion bodies containing nuclear FUS (Mackenzie et al., 2010). More than 50 mutations have been found in the *FUS* gene in ALS patients. Most of them are dominant missense mutations clustered in or around the C-terminal nuclear localization signal (NLS) (Zakaryan and Gehring, 2006; Deng et al., 2014). In this study, we reported a 29-year-old ALS patient carrying the novel *de novo* missense *FUS* mutation c.1528A > C. This variant, located in the NLS domain, caused FUS mislocalization into the cytoplasm in cellular models and induced neural toxicity in a transgenic fly model. Aggregates of cytoplasmic FUS induce cytotoxicity (Fushimi et al., 2011; Ju et al., 2011), mitochondrial dysfunction (Deng et al., 2015, 2018; Chen et al., 2016; Stoica et al., 2016), and impaired stress granule formation (Bentmann et al., 2012; Baron et al., 2013), which contributed to the pathogenesis of ALS.

The patient carrying the p.K510Q FUS mutation had initial symptoms at around age 30 and rapid progression, similar to a previously reported patient who carried K510E FUS mutation at the same amino acid (Suzuki et al., 2010). However, an ALS patient with K510R FUS mutation was reported to have later disease onset and longer disease duration (Waibel et al., 2010). These results suggested that not only different genes but also different missense mutations in the same amino acid of a specific gene could lead to clinical heterogeneity in ALS.

We systematically analyzed a cohort of young-onset ALS patients and observed that *FUS* mutations have the highest frequency. Additionally, we reported the novel K510Q *FUS* variant, which was proven to be pathogenic in both cellular and Drosophila models. Our study uncovered a mutational spectrum in young-onset ALS, providing helpful information for genetic counseling and diagnosis in the future.

## Data Availability Statement

All data generated or analyzed during this study are included in this published article.

## Ethics Statement

The studies involving human participants were reviewed and approved by the Ethics Committee of the First Hospital of Peking University. Written informed consent to participate in this study was provided by the participants’ legal guardian/next of kin. Written informed consent was obtained from the individuals, and minors’ legal guardian/next of kin, for the publication of any potentially identifiable images or data included in this article.

## Author Contributions

JD, DH, and ZW conceived the idea, designed the studies, and supervised the project. JD and WW designed and carried out the experiments, and analyzed the data. TW and JD generated the transgenic flies. ZX, MY, HL, WZ, JL, QW, YH, YY, DH, and ZW contributed to the clinical diagnosis and biopsy of ALS patients. JD, WW, QG, DH, and ZW wrote and edited the manuscript. All authors read and approved the final manuscript.

## Conflict of Interest

The authors declare that the research was conducted in the absence of any commercial or financial relationships that could be construed as a potential conflict of interest.

## Footnotes

1 http://www.hgmd.cf.ac.uk/

2 https://www.ncbi.nlm.nih.gov/clinvar/

3 https://www.pubvar.com/

4 www.ncbi.nlm.nih.gov/projects/SNP
